# Obesity prevalence and unfavorable health risk behaviors among German kindergarten teachers: cross-sectional results of the kindergarten teacher health study

**DOI:** 10.1186/1471-2458-13-927

**Published:** 2013-10-04

**Authors:** Sascha W Hoffmann, Suzan Tug, Perikles Simon

**Affiliations:** 1 Department of Sports Medicine, Disease Prevention and Rehabilitation, Faculty of Social Science, Media and Sport, Johannes Gutenberg-University Mainz, Albert-Schweitzer-Str. 22, 55128 Mainz, Germany; 2 Department of Sports Medicine / Sports Physiology, Institute of Sport Science, University of Bayreuth, Universitätsstraße 30, 95440 Bayreuth, Germany

**Keywords:** Obesity, Habitual physical activity, Screen time activities, Kindergarten teacher, Children’s health, Perception

## Abstract

**Background:**

The aim of the study was to investigate obesity status and associated health risk behaviors in a sample of German kindergarten teachers. At present, such data are not available, despite the fact that kindergarten teachers educate children at a formative time in their lives.

**Methods:**

Kindergarten teachers aged 18–62 years (n = 313) were invited to participate in the Kindergarten Teacher Health Study (KTHS) by completing a self-reported questionnaire. We analyzed their obesity status, health risk behaviors (i.e., habitual physical activity, screen time activities, eating behavior patterns, smoking), and their general ability to identify overweight children and the associated health risks of overweight and obesity based on special age- and sex-specific silhouettes. After adjusting for covariates, bivariate correlations were conducted for associations between body mass index (BMI) and health risk behaviors, while analyses of variance (ANOVAs) were used to analyze differences of health risk behaviors between BMI groups. Logistic regression analyses were conducted to predict determinants of kindergarten teachers who did not correctly identify the overweight silhouettes and their associated physical and mental health risks. Additionally, data regarding kindergarten teachers’ weight status and smoking behavior were compared with nationally representative data from the 2009 Microcensus (n = 371310) using the Mann–Whitney U-test.

**Results:**

The prevalence rates of overweight and obesity were 41.2% and 17.9%, respectively. The prevalence of obesity was significantly higher in kindergarten teachers (p < 0.001) compared to national Microcensus data. Only 44.6% of teachers were able to identify overweight children correctly. The fact that being overweight is associated with physical and mental health risks was only reported by 40.1% and 21.2% of teachers, respectively. Older kindergarten teachers were more likely to misclassify the overweight silhouettes, while younger, normal-weight, and overweight kindergarten teachers were more likely to underestimate the associated health risks. Obese kindergarten teachers reported spending more time in front of computer and television screens than their normal-weight counterparts, especially on weekends. In addition, obese kindergarten teachers reported eating less often with their families and more frequently reported watching television during meals.

**Conclusions:**

Advanced monitoring and multifaceted interventions to improve the health behaviors of kindergarten teachers should be given high priority. Because kindergarten teachers’ behavioral modeling presumably mediates children’s health behaviors, additional research is needed about kindergarten teachers’ health and its proposed interaction with children’s health.

## Background

The prevalence rates of overweight and obesity, measured as a high body mass index (BMI), have increased worldwide during the last decades [1]. Although some studies have reported that the prevalence of obesity has remained stable or even decreased in some groups, obesity prevalence is still considerably high [2,3]. High BMI has been linked with the most frequent morbidities of western societies, such as cardiovascular diseases (CVDs), type-2-diabetes (T2D) [4], musculoskeletal disorders [5], and several types of cancers [6]. The evidence-based risk factors for overweight are psychological factors [7], genetic determinisms [8], and adverse changes in general health behaviors, such as physical activity, dietary behavior patterns, smoking, and screen time activities [9-11].

Today, young children spend a large part of their days at kindergarten [12]. In Germany, 27.6% of children under the age of 3, and 93.4% of children under the age of 6 regularly attend child day care [13]. Kindergarten teachers have a particularly high interaction with the general public [14], and due to their daily contact with children, kindergarten teachers’ health behaviors likely have a lasting effect on the general health behaviors and future lives of their kindergartners. Kindergarten teachers are qualified to work with children and primarily function as role models for children and their parents because of their pedagogical skills [15,16]. Kindergarten teachers play a key role in promoting better health behaviors among children and their parents. Recent data suggest that there is a critical age range in the development of overweight among children and adolescents. Researchers found that the prevalence of overweight predominantly increased between 5.0 and 8.5 years compared to reference data from 20 years ago. Hence, time spent in kindergarten and primary school may be important for the timing of future public health approaches [17]. From these data, we assume that kindergarten teachers may play a key role in future prevention-interventions. In Germany, legislation already states that kindergarten teachers have an educational responsibility to society. Therefore, they should have the ability to recognize overweight in children and children’s risk status for experiencing certain health problems and to assign children to appropriate prevention programs as early as possible [18].

Interestingly, despite the number of studies that have examined this population, no previous study has assessed data regarding obesity status, associated health risk behaviors, and the ability to identify childhood obesity of kindergarten teachers. Therefore, the aim of this study was to present self-reported health risk behaviors from a sample of German kindergarten teachers and to examine their general ability to identify overweight silhouettes and their awareness of overweight-related health risks.

## Methods

### Study design

The Kindergarten Teacher Health Survey (KTHS) is a community-based cross-sectional population health study of German kindergarten teachers. The KTHS collected comprehensive data about BMI status and general health patterns, such as habitual physical activity (HPA), screen time activities, and smoking behavior in a representative sample of 313 kindergarten teachers aged 18 to 62 years from a large German city (≈ 200000 residents).

To recruit study participants and to generate a high response rate, we used an active recruitment strategy described below. We obtained permission to perform the study from the department head of social issues, children, youth, school, and health of the city of Mainz in July 2009. The study was approved by the Institutional Review Board of the City of Mainz. Afterwards, we informed the kindergarten management about the study and received permission to inform staff at additional team meetings, during which we provided study information to their employees. Finally, between March and May of 2010, kindergarten teachers from all 35 public kindergartens in Mainz were invited to participate in the study. Of the 459 kindergarten teachers we contacted, 328 replied to our invitation and answered the questionnaire. Fifteen of 328 respondents did not reply to questions about body height and weight. Thus, data from 313 kindergarten teachers were used for the final data analyses (response rate of 68.3%). The 68.3% response rate is similar to the response rates reported in epidemiological literature [19,20]. The final response rate of the recruited public kindergartens was 97.1% (34 out of 35 kindergartens). The management of the kindergartens that did not participate in our study reported that their employees were not interested in the study. Participation in this study was voluntary, and all participants provided written informed consent. Data were collected using a self-administered questionnaire (a paper and pencil questionnaire). The self-constructed standardized questionnaire collected anthropometric and sociodemographic characteristics, along with health and behavior reports (i.e., HPA, smoking status, screen time activities, and selected eating behavior patterns) [20-24]. Respondents returned the completed questionnaires in sealed envelopes placed in a box for pickup by project staff. The questionnaires were numbered chronologically with special identification numbers that were used to assign the kindergarten teachers to their geographical occupational environment.

In addition, the BMI status and smoking prevalence of kindergarten teachers were compared with a representative German reference population from the 2009 Microcensus [22]. The Microcensus is a nationally representative annual survey conducted by the German Federal Statistical Office. Designed as an omnibus survey, the Microcensus contains important structural data about the population, questions about family and household context, and questions about employment, income, and education. Participation in the Microcensus is obligatory. Every fourth year, a health module is included. In 2009, questions about health-related issues were addressed to 1% of the German population (340000 households with approximately 700000 persons) by the Federal Statistical Office (DESTATIS) [22]. For the present analysis, we were interested in questions about anthropometric characteristics and smoking habits. Respondents older than 65 years were excluded to ensure that estimates from the KTHS sample would be comparable with the Microcensus estimates. Thus, the Microcensus sample includes 371310 subjects (180270 women and 191040 men). The non-response rate of the Microcensus was generally low (under 10%) [22].

### Data sources

#### Anthropometric characteristics and sociodemographic factors

KTHS participants reported their height and weight. World Health Organization (WHO) criteria were used to classify people into underweight (BMI < 18.5 kg/m^2^), normal-weight (BMI = 18.5 – 24.9 kg/m^2^), overweight (BMI = 25 – 29.9 kg/m^2^), and obese (BMI ≥ 30 kg/m^2^) categories [25]. In our sample, the sociodemographic variables of age, sex, education (secondary general school, secondary modern school, and high school graduate), and native country were included.

#### Health risk behaviors

##### Data on multidimensional health risk behaviors

HPA was assessed using the Baecke questionnaire (BAQ) [21]. The questionnaire consists of 16 items from which three meaningful factors can be distinguished. The first factor consists of aspects of occupational physical activity, the second factor consists of aspects of leisure time physical activity, and the third factor assessed sport during leisure time. All participants had to reply on a five-point scale (1 = never, 2 = seldom, 3 = sometimes, 4 = often, 5 = very often or always). Finally, three indexes were calculated and introduced as continuous variables in the analyses: a work index, a leisure time index, and a sport index. All questions were translated into German. In the original publication, the test-retest reliabilities of the work, sport, and leisure time indices were 0.88, 0.81, and 0.74, respectively.

Similar to the 2009 Microcensus, smoking status was categorized as current smoker (number of cigarettes per day) and non-smoker (including former smoker and never smoker) [22].

The questionnaire assessed screen time activity using questions about the duration of television viewing (TV) or digital versatile disc viewing (DVD) and the duration of computer use, including Internet use. Respondents reported the frequency with which they engaged in these behaviors on weekdays or during weekends using a five-point scale (1 = never, 2 = approx. 30 minutes per day, 3 = 1 to 2 h per day, 4 = 3 to 4 h per day, 5 = more than 4 h per day). This subscale has been used previously in parental questionnaires within the German Health and Examination Survey of Children and Adolescents (KiGGS) [20]. Participants were divided into two groups as follows: kindergarten teachers viewing TV less than 3 h/day and those viewing TV 3 h/day or more. Additionally, kindergarten teachers were asked about different eating behavior patterns in daily life as follows: eating with their families, eating breakfast, and watching TV during meals (1 = never, 2 = seldom, 3 = sometimes, 4 = often, 5 = always). These variables were introduced as categorical variables (never/seldom vs. sometimes/often/always) in the analyses.

#### Silhouettes

Kindergarten teachers were presented with a panel of 7 silhouettes to rate their perception of children’s weight status. The silhouettes were obtained from the study of Warschburger and Kröller [24], where sets of figures were developed to represent different age and gender groups of preschool children. According to age and sex-specific cut-offs [26], 2 silhouettes represented underweight children (3^rd^ and 10^th^ percentiles), 3 silhouettes represented children within the normal-weight range (25^th^, 50^th^, and 75^th^ percentiles), and 2 silhouettes represented an overweight and obese child (90^th^ and 97^th^ percentiles) [24]. Kindergarten teachers were required to answer the following question: “Which of the silhouettes do you think represents an overweight child?” In addition, they were questioned about their perception of the physical or mental health problems associated with being overweight (e.g., “Which silhouettes do you think have an increased risk for physical/mental health problems?”). Silhouettes above the 90^th^ percentile were defined as overweight and represented a higher health risk. Kindergarten teachers were allowed to mark more than one silhouette with a cross, and their first mark was interpreted as the lowest limit. Detailed descriptions of the silhouette evaluation process have been previously published [24].

#### Occupational social environment

Taking the inner-city occupational social environment into account, we tested the hypothesis that kindergarten teachers who work in deprived city districts were more likely to be overweight or report disadvantageous health behaviors. As part of a social area analysis of the city of Mainz conducted by Pfeiffer et al. (2005), comprehensive information about general conditions (land use, living situations, demography, budgetary structure and family, employment and income maintenance, and highly problematic groups and education) and social structures were collected from each of the 65 city districts. Based on these data, the life situation index (LSI) was created to combine all relevant characteristics into one specific scale [27]. The LSI consists of 4 thematic indices (employment/working life, education, social situation/heterogeneity, and home environment) that are weighted differently. We found a linear relationship between the characteristics that the LSI assesses and the deprivation of respondents’ geographical area of residence. “Employment and working life” was weighted at 35%, “education” was weighted at 20%, “social situation/heterogeneity” was weighted at 30%, and “housing and environment” was weighted at 15%. For the purposes of calculating the LSI, the relative deviation of the value of each district was computed from the average of all districts on a single index and was then transformed to make the four indices comparable. After this transformation, the obtained values were between negative 10 and positive 10, while zero corresponded to the average index of the whole city. The LSI scale was dichotomized so that negative values represented a negative load to the respective district, and positive values denoted low stress on the district in the various indices. Hence, the LSI may be an effective tool for demonstrating socio-economic differences within specific geographic areas of the city of Mainz.

#### Statistical analysis

Descriptive statistics were computed for individual variables, subgroups defined by BMI classifications, and both sexes. Continuous variables are presented as means (SD), and categorical variables are summarized as relative frequencies (%). The Kruskal-Wallis test, one-way ANOVA with post-hoc comparison (Bonferroni), and Chi-square tests were used to test for differences in anthropometric characteristics, HPA indices, screen time activities, eating behavior patterns, perception of weight status, and associated health risks between BMI groups. Inter-item reliability for the health risk behaviors was assessed using Cronbach’s α [28]. After adjusting for age, sex, native country, and graduation, bivariate correlation analyses were conducted to test associations between anthropometric characteristics (body height, weight, and BMI), health risk behaviors (habitual physical activity, screen time activities, and smoking status), and the occupational social environment (LSI). Pearson, Spearman, and Kendall-Tau correlation coefficients were used to test associations for statistical significance. We used the Mann–Whitney U-test to evaluate differences in the prevalence of different weight categories and smoking habits between the KTHS sample and the national sample. Statistical analyses of kindergarten teachers’ perceptions of children’s weight status were analogously employed according to Warschburger and Kröller [24]. Chi-square tests were used to examine significant group differences in the frequency of the presented silhouettes. Logistic regression analyses were conducted to provide odds ratios (ORs) and 95% confidence intervals (CIs) for the estimated variables and to predict the determinants of kindergarten teachers who did not correctly identify overweight silhouettes and their associated physical (mental) health risks. All statistical analyses were conducted using SPSS for Windows, version 19.0 (SPSS, Chicago, IL), and results were considered to be statistically significant at p ≤ 0.05.

## Results

### Sample characteristics

Table 1 summarizes relevant anthropometric and sociodemographic data and the health risk behaviors classified by BMI group. The mean respondent age was 37.2 years (SD, 10.6). Almost two-thirds (59.7%) were ages 20 to 39 years, and 93.6% were female. The majority of respondents had received a secondary school education (64.9%), and 84.7% of the respondents were native Germans. The prevalence rates of overweight and obesity were 41.2% and 17.9%, respectively. Overweight respondents were predominantly aged 30 to 39 years (12.1%), and the percentage of overweight people increased successively over the entire age range (p ≤ 0.01; data not shown). The comparison of weight categories between the KTHS sample and the Microcensus sample reveals that obesity in KTHS females (17.7%) was more prevalent than in Microcensus females (10.8%) (p ≤ 0.01; data not shown). Among men, the prevalence rates of obesity were 20.0% in the KTHS sample and 13.5% in the Microcensus sample (p ≤ 0.01).

**Table 1:** **Means** (**SD**) **and percentage values of anthropometric and sociodemographic characteristics and health risk behaviors of the KTHS 2010 study sample classified by BMI groups**

**Variables**	**Normal-weight**^**a**^**(n = 184)**	**Overweight**^**b**^**(n = 73)**	**Obese**^**c**^**(n = 56)**	**Total(n = 313)**	**Cronbach’s α**
**Age (yrs)**, mean, SD	35.6 (10.2)^1^	39.7 (11.0)	39.2 (10.9)	37.2 (10.6)	
**Height (cm)**, mean, SD	167.4 (6.7)	166.5 (8.1)	166.6 (7.2)	167.1 (7.2)	
**Weight (kg)**, mean, SD	61.2 (7.0)^2^	75.1 (8.0)	96.4 (13.1)	70.8 (15.8)	
**BMI (kg/m**^**2**^**)**, mean, SD	21.8 (1.9)^2^	27.0 (1.2)	34.8 (4.5)	25.4 (5.5)	
**Graduation**, %					
Secondary general school	6.0	11.0	17.9	9.3	
Secondary modern school	66.8	58.9	66.1	64.9	
High school graduate	14.7	13.7	7.1	13.1	
Other graduation	12.5	16.4	8.9	12.8	
**Native country**					
Germany	85.9	80.8	85.7	84.7	
Others^d^	14.1	19.2	14.3	15.3	
**Habitual Physical Activity (HPA)**, mean, SD					0.924
Work index^e^	2.8 (0.4)	2.8 (0.4)	2.8 (0.3)	2.8 (0.4)	
Sport index^f^	2.8 (0.7)^3^	2.7 (0.6)	2.5 (0.5)	2.7 (0.6)	
Leisure time index^g^	2.7 (0.7)	2.6 (0.7)	2.5 (0.6)	2.7 (0.7)	
HPA score^h^	8.5 (1.2)^4^	8.0 (1.1)	7.7 (1.2)	8.3 (1.2)	
**Smoking status**, %					0.995
Current smoker	28.8	27.4	19.6	26.8	
Non-smoker	71.2	72.6	80.4	73.2	
Incl. former smoker	15.2	19.2	28.6	19.2	
Incl. never smoker	56.0	53.4	51.8	54.0	
**Screen viewing activity**, %					
TV/DVD viewing on weekdays^i^					0.922
< 3 h/day	78.3	72.9	72.7	76.1	
≥ 3 h/day	21.7	27.1	27.3	23.9	
TV/DVD viewing on weekends^j^					
< 3 h/day	50.3	51.5	44.4	49.5	
≥ 3 h/day	49.7	48.5	55.6	50.5	
Computer use on weekdays^k^					
< 3 h/day	92.9	94.3	89.1	92.5	
≥ 3 h/day	7.1	5.7	10.9	7.5	
Computer use on weekends^l^					
< 3 h/day	87.8	82.6	72.7	83.9	
≥ 3 h/day	12.2^5^	17.4	27.3	16.1	
**Perception of weight status and associated health risks**, %					0.946
General abililty to identify overweight silhouettes^m^	44.8	46.6	41.1	44.6	
KTs who identified overweight and obese silhouettes as at risk for physical health problems^n^	38.8	35.6	50.0	40.1	
KTs who identified overweight and obese silhouettes as at risk for mental health problems^o^	20.8	19.2	25.0	21.2	
**Eating together with the family**, %^p^					-
Never/seldom	9.6	10.3	16.7	11.0	
Sometimes/often/always	90.4	89.7	83.3	89.0	
**Have breakfast**, %^q^					-
Never/seldom	15.4	12.9	18.5	15.4	
Sometimes/often/always	84.6	87.1	81.5	84.6	
**Television use during meals**, %^r^					-
Never/seldom	50.3	50.7	39.6	48.5	
Sometimes/often/always	49.7	49.3	60.4	51.5	

SD = standard deviation; Yrs. = years; KTs = kindergarten teachers.

^a^BMI 18.5 - 24.99 kg/m^2^.

^b^BMI 25–29.99 kg/m^2^.

^c^BMI ≥ 30 kg/m^2^.

^d^Other countries included Turkey, Poland, Russia, and Kazakhstan.

^e^n = 261.

^f^n = 260.

^g^n = 238.

^h^n = 193.

^i^n = 305.

^j^n = 303.

^k^n = 307.

^l^n = 304.

^m^n = 312.

^n^n = 312.

^o^n = 312.

^p^n = 299.

^q^n = 306.

^r^n = 303.

^1^P ≤ 0.05 for ANOVA with post hoc comparison using Bonferroni adjustment for differences between the normal-weight and overweight category.

^2^P ≤ 0.01 for ANOVA with post hoc comparison using Tamhanes-T2 adjustment for differences between all BMI categories and weight categories.

^3^P ≤ 0.01 for ANOVA with post hoc comparison using Bonferroni adjustment for differences between the normal-weight and obesity categories.

^4^P ≤ 0.01 for ANOVA with post hoc comparison using Tamhanes-T2 adjustment for differences between the normal weight and obesity categories.

^*5*^P ≤ 0.05 for Chi-square tests for differences between the BMI categories.

### Health risk behaviors

Table 1 also shows the health risk behaviors of the study respondents by BMI group. Internal consistency was assessed by Cronbach’s α. All outcomes were acceptable: HPA levels (α = 0.924), smoking (α = 0.995), screen time activities (α = 0.922), and perception of weight status (α = 0.946). In terms of general health risk behaviors, normal-weight kindergarten teachers participated in fewer screen time activities during their leisure-time than their overweight or obese counterparts. Moreover, overweight and obese kindergarten teachers were more likely to use the computer on weekends for more than 3 h/day compared to normal-weight kindergarten teachers (p ≤ 0.05). In summary, participants were more likely to participate in more screen time activities on weekends than on weekdays. Normal-weight participants had significantly higher total HPA scores than obese participants (p ≤ 0.01). This observation also applied to the sport index (p ≤ 0.01), while no significant differences were observed in the three activity indices between males and females (data not shown). Overall, 64% of the respondents were physically active at least once a week. Conversely, 60% of the underweight, 26% of the normal-weight, 43% of the overweight, and 50% of the obese participants were not physically active. Obese kindergarten teachers were more likely to be former smokers than their overweight and normal-weight counterparts. Obese kindergarten teachers were less likely to smoke compared to normal-weight or overweight kindergarten teachers. Overall, kindergarten teachers were less likely to be smokers than the German population. However, statistically significant differences were only noted in males and not in females (p ≤ 0.01; data not shown). One-third of KTHS males were current smokers at the time of the study, and 70.0% were non-smokers, compared to 37.8% (smokers) and 62.2% (non-smokers) in the Microcensus sample. In addition, we analyzed eating behavior patterns based on BMI group. We found that obese kindergarten teachers eat with their family or have breakfast less often and use the television during meals more than normal-weight kindergarten teachers. No sex and BMI group differences were observed.

The bivariate correlations between the KTHS sample’s anthropometric characteristics and health risk behaviors are shown in Table 2. There were significant associations between BMI and HPA scores (p ≤ 0.001) and between BMI and smoking behavior (p ≤ 0.05). Furthermore, BMI was inversely associated with the occupational social environment (classified as LSI; p ≤ 0.05), which is characterized by high area-level deprivation. Similarly, we found an inverse association between computer use on weekends and working in a deprived city district. However, positive correlations were identified between body weight and TV/DVD viewing on both weekdays (p ≤ 0.05) and weekends (p ≤ 0.01), while the sport and leisure time indices were inversely correlated with TV/DVD viewing on both weekdays (p ≤ 0.01) and weekends (p ≤ 0.05). No statistical relationship was found when eating behavior patterns were included in the final correlation analysis.

**Table 2:** **Correlations between anthropometric characteristics**, **health risk behaviors**, **and the occupational social environment of the total KTHS study sample**

**Attributes**	**Anthropometric characteristics**			**Physical activity**				**Screen time activities**				**Smoking status**	**Occupational social environment**
	**BMI(kg/m**^**2**^**) (n = 313)**	**Weight (kg) (n = 313)**	**Height (cm) (n = 313)**	**Work index (n = 261)**	**Sport index (n = 260)**	**Leisure time index (n = 238)**	**HPA score (n = 193)**	**TV/DVD viewing, weekdays (n = 313)**	**TV/DVD viewing, weekends (n = 313)**	**Computer use,weekdays (n = 313)**	**Computer use,weekends (n = 313)**	**Smoking s (n = 313)**	**LSI (n = 313)**
**Anthropometric data**													
BMI (kg/m^2^)	1.000	0.897***^b^	-0.129*^b^	-0.026^b^	-0.124*^b^	-0.075^b^	-0.277***^a^	0.065^c^	0.079^c^	0.043^c^	0.069^c^	0.148**^a^	-0.128*^b^
Weight (kg)		1.000	0.277***^b^	-0.029^b^	-0.115^b^	-0.073^b^	-0.257***^a^	0.094**^c^	0.113**^c^	0.056^c^	0.052^c^	0.171**^a^	-0.086^b^
Height (cm)			1.000	0.048^b^	0.030^b^	0.024^b^	-0.009^a^	0.037^c^	0.062^c^	0.030^c^	-0.047^c^	0.067^a^	0.044^b^
**Habitual physical activity**													
Work index				1.000	0.067^b^	0.074^b^	0.427***^a^	0.040^c^	0.072^c^	0.003^c^	0.043^c^	-0.113*^a^	-0.094^b^
Sport index					1.000	0.289***^b^	0.761***^a^	-0.127*^c^	-0.099*^c^	-0.033^c^	-0.087^c^	0.040a	0.001^b^
Leisure time index						1.000	0.768***^a^	-0.193***^c^	-0.196***^c^	-0.074^c^	-0.177***^c^	-0.001^a^	-0.014^b^
HPA score							1.000	-0.158**^c^	-0.140**^c^	-0.057^c^	-0.141**^c^	-0.075^a^	-0.063^a^
**Screen time activities**													
TV/DVD viewing, weekdays								1.000	0.478***^c^	0.149***^c^	0.189***^c^	0.063^c^	-0.033^c^
TV/DVD viewing, weekends									1.000	0.096*^c^	0.218***^c^	-0.010^c^	-0.004^c^
Computer use, weekdays										1.000	0.532***^c^	-0.062^c^	0.001^c^
Computer use, weekends											1.000	-0.130**^c^	-0.121**^c^
**Smoking status**													
Smoking												1.000	0.056^a^
**Occupational social environment**													
LSI													1.000

*P ≤ 0.05; **P ≤ 0.01; ***P ≤ 0.001.

BMI = Body mass index; HPA = Habitual physical activity; LSI = Life situation index.

^a^Pearson coefficient; adjusted for age, sex, native country, and graduation.

^b^Spearman-Rho coefficient; adjusted for age, sex, native country, and graduation.

^c^Kendall-Tau coefficient; adjusted for age, sex, native country, and graduation.

### Influences of the general ability to identify overweight silhouettes and the awareness of overweight-related health risks

In total, 44.6% of the respondents correctly identified silhouettes associated with overweight in children (Table 1). We further analyzed the influence of variables such as sex, age, LSI, BMI, educational level, native country, eating behavior patterns, habitual physical activity, and screen viewing activities on teachers’ general ability to identify overweight silhouettes and their associated health risks (see Table 3). No differences were observed between kindergarten teachers who correctly identified overweight silhouettes and teachers who did not with regard to sex, LSI, BMI, educational level, native country, smoking status, eating behavior patterns, habitual physical activity, and screen time activities. Older kindergarten teachers correctly identified the overweight silhouettes significantly less frequently than their younger colleagues. Only 40.1% indicated that the overweight silhouettes were at risk for physical health, and even fewer (21.2%) associated overweight and obesity with mental health problems. More often than their counterparts, normal-weight, overweight, and younger kindergarten teachers did not identify increased risks for physical and mental health problems associated with overweight.

**Table 3:** **Factors influencing general ability to identify overweight silhouettes and the awareness of overweight**-**related health risks**^**a**^

	**Correct**	**Incorrect**	p*	**OR**^**b**^	**95%CI for OR**^**c**^
**The influence of age on general ability to identify overweight silhouettes**					
**Age group**					
18-29	43	47	**0.041**	0.51	0.25 - 1.03
30-44	69	72		**0.45**	**0.24** - **0.85**
45-65	27	54		1.00	
**The influence of BMI on the awareness of overweight**-**related physical health risks**					
**BMI group**					
Normal-weight	71	112	**0.022**	**2.08**	**1.05 - 4.11**
Overweight	26	47		**2.43**	**1.10 - 5.38**
Obese	28	28		1.00	
**The influence of age on the awareness of overweight**-**related mental health risks**					
**Age group**					
18-29	13	77	**0.028**	**2.85**	**1.21 - 6.72**
30-44	28	113		**1.97**	**0.96 - 4.04**
45-65	25	56		1.00	

^a^Only significant associations are displayed.

^b^OR = Odds ratios are given for the probability of an incorrect perception considering the first parameter.

^c^ORs and 95% confidence intervals (CIs) are from multiple logistic regression analyses in which all independent variables were included simultaneously: sex, LSI, BMI group, age group, educational level, native country, smoking status, eating behavior patterns (eating with the family, eating breakfast, TV use during meals), HPA, TV/DVD viewing on weekdays and on weekends, and computer use on weekdays and on weekends.

*P < 0.05; significance in bold print.

## Discussion

Although current obesity trends in central Europe and the US are stable or decreasing [2,29], our results demonstrate that German kindergarten teachers show statistically significantly higher rates of obesity compared to a representative German reference population. We found that kindergarten teachers have a statistically significant increased risk for obesity if they are less physically active, have high screen time activities, and work in socially deprived city districts.

It has already been reported that certain sub-populations with specific occupations, such as unskilled workers, firefighters, motor vehicle operators, and health technicians or other health service occupations, have an increased risk for obesity [30-36]. Some studies have reported sex-specific influences in occupational class and the development of overweight and obesity [35,36]. The HPA indices we observed in the obese participants nicely reflect the results of the NUGENOB (NUtrient-GENe interactions in human OBesity) study [37]. The results of the NUGENOB study indicate that there is a statistically significant correlation between a general obesity index and the HPA score (p < 0.0001). Our results regarding an association between HPA score and overweight in German kindergarten teachers echo this finding. Correlation analyses indicate that all kindergarten teachers seemingly participate in more screen time activities and less habitual physical activity during their leisure time, particularly on weekends. Additional analysis reveals that when the respective response rate of a kindergarten is lower compared to other kindergartens, kindergarten teachers from these kindergartens participate in more screen time activities during their leisure time than teachers from kindergartens with higher response rates (data not shown). In this context, this finding suggests that social desirability may have influenced these data.

Obese kindergarten teachers in the group with the highest computer use (≥ 3 h/day) used the computer on weekends significantly more often than their normal-weight colleagues. Similar to this finding, recent data suggest that leisure time sedentary behavior (based on TV viewing) is independently associated with mental health in adulthood and that sedentary behavior should be addressed as an independent health risk [38]. The overall associations between screen time activities, physical activity, and weight status in this study are consistent with the associations identified in previous studies [11,38]. Previous research also suggests that TV use is associated with increased dietary intake and high BMI, particularly among women [39]. We found that the prevalence of TV use during meals is highest among obese kindergarten teachers. Although we did not observe a statistical association between eating behavior patterns and overweight in kindergarten teachers, further attention should be given to the implementation of workplace health promotion and child health promotion interventions. A link between daily TV use during meals and higher BMI among pre-school children has already been reported by Dubois et al. [40]. They concluded that health professionals (kindergarten teachers) should help families to find strategies to reduce daily TV use during meals. However, because our findings show that kindergarten teachers themselves have similar behavior patterns, this suggestion may be unrealistic. The high prevalence of (obese) kindergarten teachers who do not have breakfast is also of high interest for overweight and obesity prevention. Cho et al. suggested that having breakfast is associated with lower BMI in adults [41]. Previous research also found that, relative to a control group, individuals who skip breakfast were more likely to be overweight or have abdominal obesity, elevated blood pressure, elevated total serum cholesterol or elevated serum insulin [42]. Future intervention strategies with kindergarten teachers should consider these issues.

Additionally, we found that kindergarten teachers have a lower smoking prevalence than the general German population. These findings echo a study conducted by Ohida et al., who found similar smoking prevalence results within a large cohort of Japanese kindergarten and schoolteachers and the general Japanese population, though the total smoking prevalence is higher in Japan [43].

The present study was a first attempt to investigate whether the obesity status of kindergarten teachers may be affected by the social area in which they are employed. We identified an inverse association between kindergarten teachers’ BMIs and their occupational social environments, which is characterized by high social area-level deprivation in the respective city districts. To our knowledge, this study is the first to investigate the burden of obesity across the occupational social environment of kindergarten teachers. However, previous research has reported that high BMI in adolescents and adults was associated with higher social area-level deprivation [44,45]. In addition, another study from the UK found evidence for area-level socio-economic inequalities in the prevalence of non-communicable chronic diseases, such as chronic kidney diseases [46].

While more is known about parents’ perception of their own child’s weight status [47-52], there is a dearth of literature that examines the general accuracy of parents’ perception of children’s weight status [24,53]. To date, nothing is known about kindergarten teachers’ general ability to correctly identify overweight children and their associated health risks. In this study, over half of the sample incorrectly identified overweight silhouettes. Nearly 60% were unable to identify overweight silhouettes as at risk for physical health problems, and nearly 80% did not identify the increased mental health risks associated with overweight or obesity. We found no sex, weight, or demographic-related group differences in kindergarten teachers’ perception of children’s weight status. Older kindergarten teachers failed to identify overweight in children, while normal-weight, overweight and younger kindergarten teachers were less frequently aware of the associated physical and mental health risks. The general ability to identify overweight silhouettes and to associate silhouettes above the 90^th^ percentile with a higher risk for physical and mental health problems was lower compared to the sample in the Warschburger and Kröller study [24]. Interestingly, obese kindergarten teachers are more aware of physical and mental health risks than those who are overweight. In a recently published study, West et al. found that people who classified themselves as obese may more accurately identify obese children than people who described themselves as overweight or normal-weight [54]. We believe that obese kindergarten teachers have a previously developed awareness of obesity-related physical and mental health problems, possibly because obese kindergarten teachers have had personal experiences with weight-related physical comorbidities or psychological strains [4,5,55,56]. Compared to other studies, our study found that kindergarten teachers have a lower awareness of the presence of overweight in children and its association with physical or mental health risks [24,53]. Future longitudinal studies should investigate whether and to what extent these adverse conditions and health behaviors affect children’s physical and mental development. Recent data suggest that the transition between kindergarten and primary school may be associated with the development of overweight in children [17]. Therefore, our findings may have important public health implications. Designing interventions targeted at kindergarten teachers is a meaningful approach to improving public health. Our findings also suggest that the potential exists to improve awareness among kindergarten teachers through special skill enhancement programs. At present, such programs do not exist and must be developed. Therefore, we recommend evaluating the training that German kindergarten teachers receive because currently, no comprehensive health module is included. Whether restructuring the training program would successfully increase kindergarten teachers’ awareness of children’s weight status must be investigated in further longitudinal studies. Even without the existence of such programs, kindergarten teachers should recognize that overweight and obesity is already present in kindergartners and poses a significant health risk to their future lives [57]. Regular examinations of kindergartners’ anthropometry and motor skills will aid in the early detection of negative developments and may support the German medical health examination system. Given the long-term trust established with parents, kindergarten teachers could get in touch with “high-risk families” and give feedback about children’s physical health status and eating behaviors.

Thus, we support improvements in kindergarten teacher training as a means of enhancing their role as future health educators. Kindergarten teachers need to possess good physical and mental health to meet the care requirements of their students. Hence, occupational health in this working population can play a decisive role in future prevention interventions, which should include a focus on both the occupational environment and individual behaviors [30].

The findings of this study are subject to the limitations of respondent self-reporting and its cross-sectional design, which limits our ability to draw conclusions about the associations between exposures and outcomes. Causal relationships are not clear at present. However, for the first time, this study attempts to characterize obesity status and health risk behaviors in a German kindergarten teacher population. Because we used established scales to measure overweight and obesity, we were able to conduct a large number of analyses to confirm associations between variables in our cohort. Because of the confirmatory nature of these analyses, we are confident that our observed outcomes were not biased by chance. Our finding that kindergarten teachers do not have advanced skills to recognize obesity or advanced knowledge about obesity is in line with other groups at risk for obesity. However, kindergarten teachers must recognize weight gain in their kindergartners.

To gain more insight into causal relationships and to assess the independent impact of risk factors on obesity, other types of studies are needed. Our data suggested that kindergarten teachers’ obesity status may be associated with deprived social area-levels in the city districts of Mainz, but we do not know if these results can be confirmed in other cities. Unfortunately, we were unable to measure additional individual deprivation markers, such as home and neighborhood environment. In addition, further attention should be given to the fact that the use of self-reported weight and height could lead to underestimates of obesity prevalence because subjects tend to underreport their body weight [58-60], especially obese subjects [60,61]. This possibility should be taken into consideration when using data to make decisions concerning public health recommendations [62].

The main strengths of this study are its large sample size of kindergarten teachers from a representative local area with newly collected data of anthropometric and sociodemographic characteristics, along with HPA, screen time activities, and smoking habits. Moreover, comparing kindergarten teachers’ BMI status and smoking prevalence with a nationally representative German sample gave us the opportunity to quantify the increased obesity incidence in the occupational group of kindergarten teachers. Additionally, for the first time, we provide data about the teachers’ perception of children’s weight status and its association with physical and mental health risks.

## Conclusions

The KTHS should provide statistical evidence for prevention interventions designed to change kindergarten teachers’ daily health behaviors, with a particular focus on increasing daily physical activity over a period of time. Data from other countries do not exist at present. Our findings suggest that kindergarten teachers were not able to recognize overweight and obesity in children. Therefore, further research should focus on evaluating the effects of restructuring the teacher training programs and starting personal lifestyle counseling for health risk behaviors. Using body composition measurements in combination with objectively measuring total energy expenditure will help to quantify the results of this study. Randomized-controlled trials with sufficient power and suitable outcome measurements are needed for future evaluations. Because kindergarten teachers’ health behaviors may act as primary mediators for children’s health behaviors, additional research is needed to study kindergarten teachers’ health and its proposed influence on children’s weight and physical activity status.

## Abbreviations

BAQ: Baecke Questionnaire; BMI: Body mass index; CVD: Cardiovascular disease; DESTATIS: Federal Statistical Office; DVD: Digital versatile discs; H: Hours; HPA: Habitual physical activity; KiGGS: German Health and Examination Survey of Children and Adolescents; KTHS: Kindergarten Teacher Health Study; KTs: Kindergarten teachers; LSI: Life situation index; T2D: Type 2 diabetes; TV: Television; WHO: World Health Organization.

## Competing interests

The authors declare that they have no competing interests.

## Authors’ contributions

SWH was involved in study design, data collection, and data analysis and drafted the final version of the manuscript. ST was involved in data collection and also drafted the manuscript. PS is the principal investigator of the KTHS, contributed to the study design, and was also involved in statistical data analysis and writing the final version of the manuscript. All authors listed approved the final version of the manuscript.

## Pre-publication history

The pre-publication history for this paper can be accessed here:

http://www.biomedcentral.com/1471-2458/13/927/prepub
